# Factors contributing to medication safety incidents observed by community pharmacists - a content analysis of incident reports

**DOI:** 10.1016/j.rcsop.2026.100851

**Published:** 2026-09-21

**Authors:** Milka Lohtaja, Anna Schoultz, Iina Mönkkönen, Anna-Riia Holmström

**Affiliations:** a Division of Pharmacology and Pharmacotherapy, Faculty of Pharmacy, University of Helsinki, Helsinki, Finland; b Pharmacy Kaari, Kantelettarentie 1, 00420 Helsinki, Finland; c Faculty of Veterinary Medicine, University of Helsinki, P.O. Box 66 (Agnes Sjöbergin katu 2P), 00014 Helsinki, Finland

**Keywords:** Community pharmacists, Medication error, Contributing factors, Medication error reporting, Medication safety, Outpatient care

## Abstract

**Background:**

Medication errors pose a risk to outpatient medication safety. Community pharmacists play a central role in medication safety; however, their contribution to risk management in social and healthcare settings is less studied.

**Objectives:**

This study aims to explore factors identified by community pharmacists contributing to medication errors originating outside pharmacies. It aims to show how community pharmacies can collaborate with social and healthcare organisations to enhance medication safety.

**Methods:**

Medication safety incident reports from three wellbeing services counties in Finland were analysed using qualitative content analysis. A data-driven approach with the Systems Engineering Initiative for Patient Safety (SEIPS) model was applied to categorise and quantify contributing factors in incident reports.

**Results:**

All detected incidents (*n* = 461) concerned prescribing errors, with contributing factors identified in 80% (*n* = 367/461). Altogether, contributing factors were identified 573 times in the incident reports. Organisational factors were the most common, constituting deficiencies in written communication (14%, *n* = 81/573) and in work environment and management (13%, *n* = 76/573). These were followed by task-related factors comprising deficiencies in working methods (15%, *n* = 88/573), and person-related factors, (e.g., lack of knowledge and skills) (8%, *n* = 48/573). The least emerging factors were related to physical environment (<1%, *n* = 1/573), technical problems in prescription writing (1%, *n* = 6/573), and packaging or features of medicinal products (2%, *n* = 11/573).

**Conclusion:**

Community pharmacists were able to identify factors behind prescribing errors from other social and healthcare organisations. Their insights can help improve prescribing processes and medication safety, supporting the value of inter-organisational collaboration, though further research and standardised reporting are needed.

## Highlights

• Community pharmacists identified factors contributing to medication errors originating in other healthcare organisations.
• Organisational factors were the most common contributors to medication errors.
• Risk information from community pharmacies can improve prescribing practices and medication risk management.
• Inter-organisational medication error reporting between community pharmacies and healthcare organisations promotes safety.

## 1 Introduction

Medication errors are a significant global health concern, affecting an estimated 1.3 million people annually.^1^ These errors can occur at any stage of the medication management and use (MMU) process, including prescribing, dispensing, administration, or monitoring.^2^, ^3^, ^4^ According to the current evidence, 53% of the harm arises during prescribing and 36% during treatment monitoring and reporting, with one in four errors resulting in severe or life-threatening patient outcomes.^4^ Given the magnitude of harm associated with prescribing, this stage represents a particularly critical point in the MMU process.^4^, ^5^ Although previous research has explored the incidence and prevalence of prescribing errors,^6^, ^7^, ^8^ evidence has largely been limited to quantitative studies describing error rates and frequencies. Research investigating factors contributing to prescribing errors has primarily been conducted in inpatient settings, where communication failures, deficiencies in patient information, workflow-related challenges, and system-level issues have been identified as important contributors.^9^, ^10^, ^11^, ^12^, ^13^ In contrast, evidence on the factors underlying prescribing errors in outpatient care remains limited, particularly from the perspective of community pharmacists. Consequently, considerably less attention has been devoted to understanding the underlying circumstances behind errors. As a result, contributing factors to prescribing errors in outpatient care remain largely underexplored.

A comprehensive understanding of these factors is essential for effective medication risk management in outpatient settings.^14^, ^15^, ^16^, ^17^ Although many countries have established reporting systems that collect incident data from both hospital and community-based care providers^,^ the data are not yet systematically used to prevent medication errors at different stages of the outpatient MMU process.^18^, 19., 20 Further research is needed to identify these factors and translate them into practical interventions, safeguarding the patients in outpatient care. To address the present evidence gap, various methodologies, such as qualitative interviews and observational studies, can be applied.^17^, ^21^, ^22^, ^23^ Especially, analysis of open narrative data from incident reports has proven a valuable approach.^18^, ^21^, ^24^, ^25^ Studies often apply theoretical frameworks such as Reason's Accident Causation Model, which categorises factors into active failures, error-producing conditions and latent conditions.^26^, ^27^ Other widely used approaches include the Framework for Analysing Risk and Safety in Clinical Medicine^28^, ^29^ and the WHO Conceptual Framework for Patient Safety.^30^ The Systems Engineering Initiative for Patient Safety (SEIPS) model, in particular, provides a systems-oriented approach by examining how people, tools, processes, and environments interact to influence treatment outcomes.^31^, ^32^, ^33^, ^34^ Despite these frameworks, inconsistencies in classification systems, methodologies, and study quality continue to present challenges in outpatient research context.^17^, ^22^, ^35^

Managing medication risks in outpatient care is inherently multidisciplinary and requires examining risk points and contributing factors from various professional perspectives. Community pharmacists, who routinely identify and mitigate prescribing errors during dispensing, hold a key position in this process.^36^, ^37^ However, collaboration between pharmacists and other healthcare providers remains limited in many countries.^38^, ^39^, ^40^, ^41^, ^42^ As a result, the potential of community pharmacies to contribute to understanding and preventing prescribing errors is often underutilised, representing an important target for health services research. This study aimed to explore the factors identified by community pharmacists as contributing to prescribing errors, based on their observations during routine dispensing activities. Understanding these factors may help generate system-level knowledge on risk points in the outpatient MMU process and inform collaborative medication safety initiatives across healthcare settings.

The study was conducted in Finland, where the current electronic patient safety incident reporting system enables shared, systems-based reporting and learning between community pharmacies and other healthcare organisations. Previous Finnish studies based on voluntary incident reporting have demonstrated substantial numbers of medication error reports across the MMU process.^43^, ^44^, ^45^, ^46^, ^47^ Pharmacists have reported approximately 400 prescribing errors per month in Finnish community pharmacies, and a total of 19,550 dispensing errors were reported between 2015 and 2020.^43^ Despite the considerable volume of reported medication errors, research evidence on prescribing errors and the factors contributing to them remains limited, particularly in outpatient care and community pharmacy settings.

## 2 Materials and methods

### 2.1 Study context

#### 2.1.1 Medication prescribing and dispensing in Finnish social and healthcare settings

Finland has a public healthcare system complemented by private and occupational healthcare services.^48^, ^49^ Twenty-one well-being service counties, along with the capital Helsinki, are responsible for providing primary and secondary care, in conjunction with university hospitals that offer tertiary care.^49^ The social and healthcare system also includes services such as maternity and child health clinics, school and student health care, oral health, mental health and substance abuse care, rehabilitation, and elderly services. Medications can be prescribed by licensed physicians and, under supervision, by trained nurses authorized to prescribe a limited range of medications, including selected antibiotics (e.g., nitrofurantoin and chloramphenicol), vaccines, and cardiovascular medications (e.g., bisoprolol and amlodipine).

In addition to the social and healthcare system, Finland has over 800 community pharmacies that are responsible for the retail distribution of prescription-only and self-care-intended over-the-counter (OTC) medicines.^50^ These pharmacies dispense 85 million prescriptions annually.^51^ The number and location of pharmacies are regulated by the Finnish Medicines Agency (Fimea) to ensure nationwide, equal access to pharmacy services.^52^ Prescriptions that community pharmacists dispense are issued electronically by various social and healthcare organisations, including primary care, outpatient, and inpatient settings, with the majority of patients being covered by primary care. Pharmacists are responsible for ensuring medication safety, and only licensed professionals are authorized to dispense prescriptions. Community pharmacists dispense medications to care homes and home care services and implement automated dose-dispensing (ADD). Specific characteristics of the Finnish prescribing and dispensing process that influence the implementation of medication safety are presented in Additional file 1.

#### 2.1.2 Inter-organisational patient safety incident reporting

In Finland, voluntary incident reporting is the primary method for social and healthcare organisations to obtain information on medication errors and their contributing factors. The electronic Patient Safety Incident Reporting System (HaiPro), introduced in 2007, supports anonymous, voluntary, and confidential reporting. It has since been adopted by over 200 organisations across various sectors of social and healthcare services.19., 44

In 2021, community pharmacies began using the HaiPro system to report medication safety incidents related to prescribing, dispensing, and OTC medications.^53^ The system currently enables inter-organisational reporting between pharmacies and other social and healthcare units [Fig. 1]. Incident reports can be submitted by any healthcare professional working in a community pharmacy, including pharmacists and other pharmacy staff. The HaiPro reporting database includes a variable identifying the reporter's profession, which can be used in research to identify reports submitted by specific professional groups, such as community pharmacists.^19^ The reporting system uses a combination of structured drop-down menus with predefined categories and free-text fields where reporters can describe the incident in their own words. This approach enables the collection of both standardised and detailed information, allowing incident reports to capture the circumstances and characteristics of each incident as accurately as possible. When pharmacists detect incidents originating in other units, they can report them through HaiPro to the respective unit, and, in turn, care units can report community pharmacy-related incidents, such as errors in medication counselling.^54^ This bidirectional reporting facilitates the sharing of safety data, collaborative risk management, and the continuous improvement of medication processes. By capturing incidents throughout the outpatient MMU process, the system supports the development of a safer, more integrated medication-management framework in Finland.

**Fig. 1:**
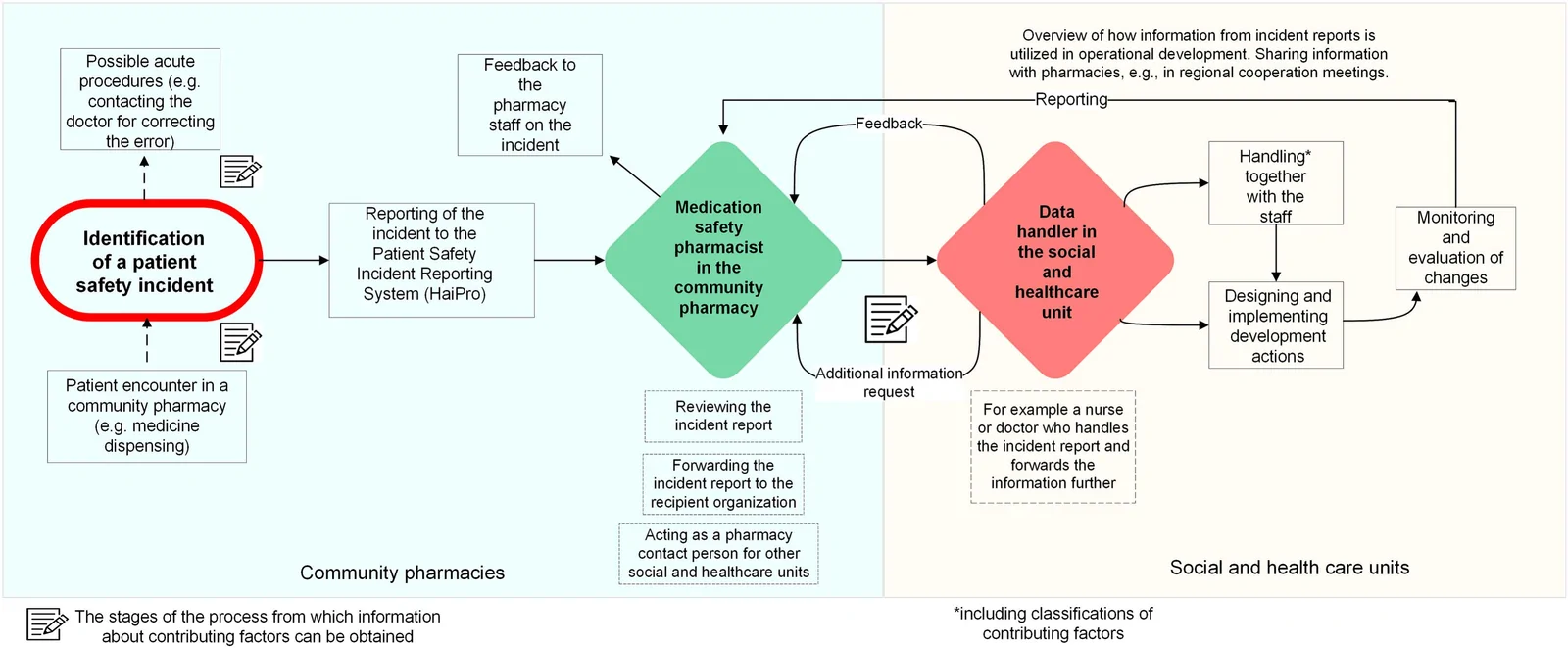
Inter-organisational patient safety incident reporting process between community pharmacies and other social and healthcare units in Finland (modified from the National Medication Safety Programme of Community Pharmacies in Finland (Valo).^54^

### 2.2 Study design and theoretical framework

This study was a qualitative retrospective register-based study. The study described factors contributing to medication safety incidents that originated in social and healthcare units but were identified and reported by community pharmacists. In community pharmacies, information about contributing factors is traditionally obtained from the patients during prescription dispensing or directly from the physicians when handling an occurred error [Fig. 1]. Additionally, pharmacies receive information through incident reports submitted to the Haipro system when the data handler of the social and healthcare organization (typically a unit nurse or doctor) supplements the report with the organization's perspective on the incident. The study employed medication safety incident reports from three wellbeing services counties in Finland between February 1, 2022, and December 31, 2023. The selected wellbeing services counties were included because they were the only regions where medication incident data could be shared between community pharmacies and other healthcare units at the time of the study. This cross-organisational information-sharing practice was newly implemented, and these counties were actively piloting the collaborative reporting process with community pharmacies when the data were extracted. The combined population of these three wellbeing services counties was approximately 553,000 inhabitants, representing about 11% of the Finnish population.

The study's theoretical framework was the human error theory, widely used in medication safety research within social and healthcare settings.^26^ According to this theory, errors are not primarily the result of individual actions but often arise from systemic issues within organisations. The theory emphasises that preventing errors involves addressing system elements as part of a systems-thinking approach. This study applied the SEIPS model, expanded from human error theory, to evaluate systemic factors of medication safety incidents.^31^ It has been effectively utilised across the healthcare sector to design safer systems, evaluate processes, and identify opportunities for improvement that can reduce errors and enhance patient outcomes.^55^, ^56^

### 2.3 Data processing

The original data of the present study comprised 457 medication-related incident reports reported by community pharmacists. The data were provided by the Association of Finnish Pharmacies in Microsoft Excel format in January 2024 (see Declarations; I. Ethics of experimentation and informed consent). The data were manually reviewed by two researchers (ML, IM), both licensed pharmacists with several years of professional experience in medication safety and risk management, to identify any technical errors or incomplete reporting in the incident reports. They were corrected in cases where obvious mistakes or deficiencies were identified. They were corrected in cases where obvious mistakes or deficiencies were identified [Fig. 2]. Based on the incident descriptions, cases that were not medication safety incidents occurring within social and healthcare units were excluded from the data (*n* = 11). Incident reports that included two or more incidents reported as one were divided into separate cases (*n* = 15). Consequently, the final dataset comprised 461 medication-related incident reports, which were included in the analyses. After correcting the data, the open narrative sections of reports that included information on contributing factors or incident circumstances were included.

**Fig. 2:**
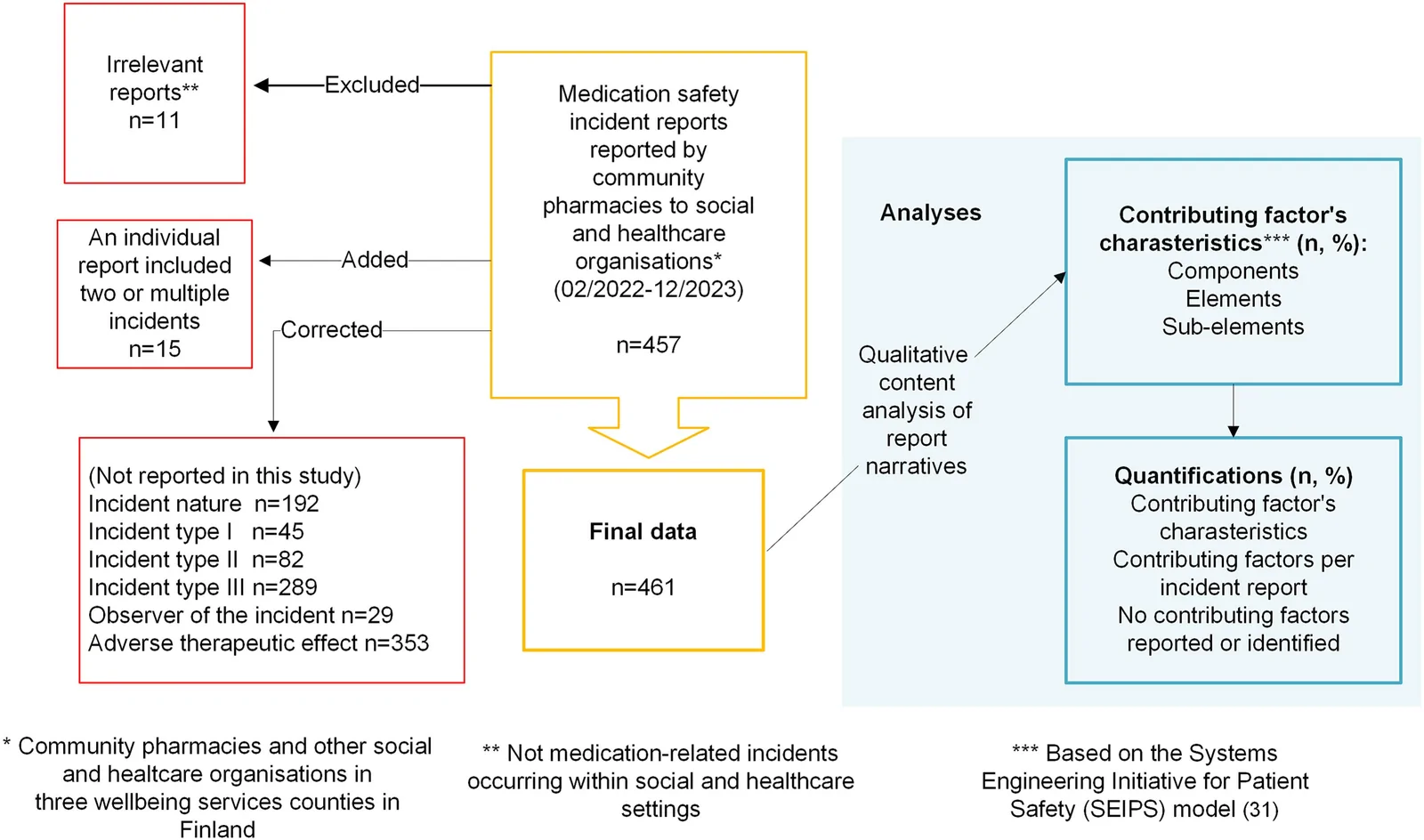
Compiling and processing of the data and the subsequent analyses.

### 2.4 Data analysis

A qualitative content analysis of incident reports with an abductive approach was conducted using Atlas.ti software, with a subsequent descriptive quantification. The coding and grouping of the data were conducted by one researcher (ML). First, contributing factors were classified in a data-driven manner^57^; passages describing contributing factors were coded from the data, after which codes with similar content were combined into a single sub-element, constituting one contributing factor. Subsequently, the sub-elements were classified under elements that were derived from the SEIPS model. Finally, the elements were organised under components aligned with the SEIPS model. An example of the qualitative content analysis is shown in Fig. 3 to demonstrate the analysis. In addition, an example from the data was provided for each sub-element to illustrate the validity of the analysis [Table 1]. A descriptive quantification of the data was performed using Microsoft Excel. The results were presented as frequencies (numbers and percentages) [Fig. 4; Table 1].

**Fig. 3:**
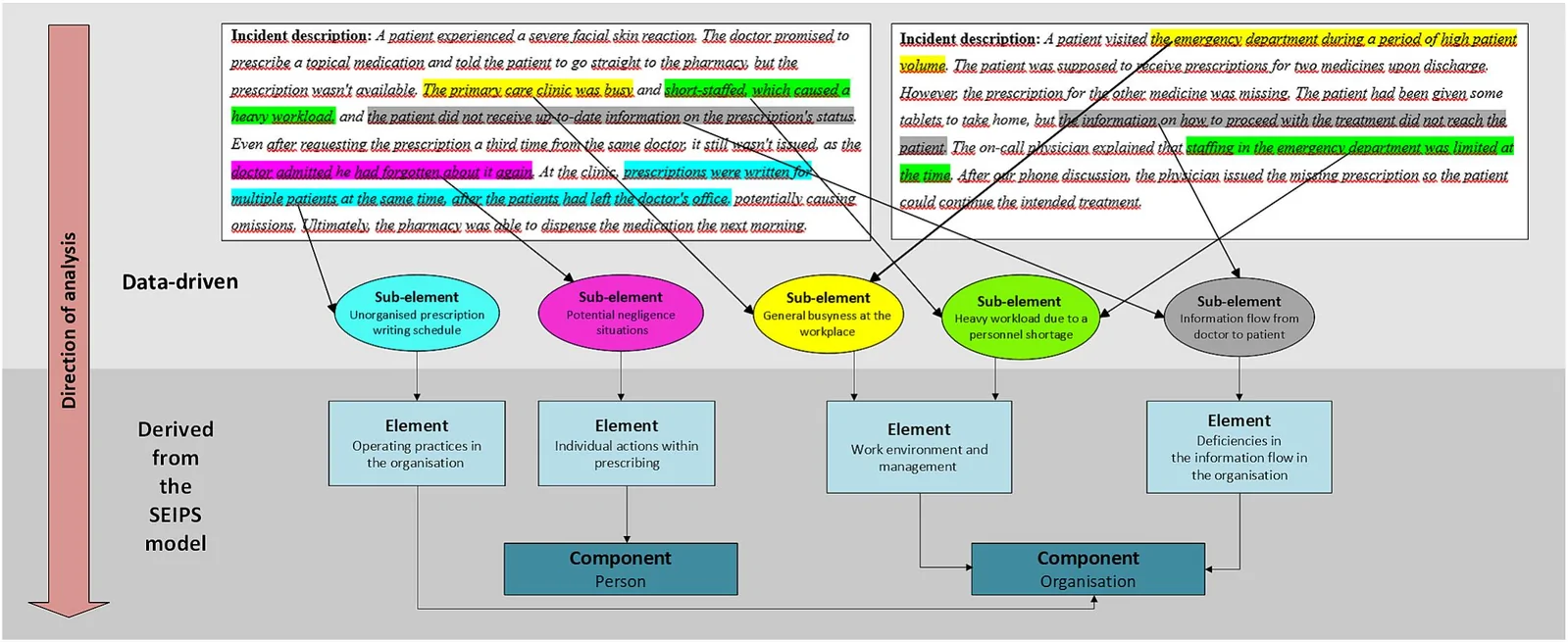
Demonstration of the qualitative content analysis of incident reports to identify contributing factors of medication errors. Sub-element representing one contributing factor, and element representing a group of contributing factors. Elements were organised under components aligned with the SEIPS model (the Systems Engineering Initiative for Patient Safety model).^31^

**Table 1:** A detailed description of contributing factors in medication safety incidents categorised based on the Systems Engineering Initiative for Patient Safety (SEIPS) model.^31^ Numbers (n) and percentages (%) indicate the frequency of the contributing factors in the data (*n* = 573).

Components	Elements (n, %)	Sub-elements (n, %)	Example from the data⁎
Organization			
	Written communication (81,14%)	Missing or incorrect dosing instructions (47, 8%)	*Exemestane was prescribed with the dosage instructions “25 mg orally, single dose no more than 1 mg”.*
		Incorrectly prescribed medication, or insufficient amount of prescribed medication (26, 5%)	*The patient was prescribed apixaban instead of the intended prescription of oxycodone.*
		An unclear and outdated list of medications (8, 1%)	*The patient was prescribed digoxin, but an unclear medication list resulted in a triple dose.*
	Work environment and management (76, 13%)	General busyness at the workplace (45, 8%)	*Due to rush, the doctor did not check the patient's vaccination status and prescribed a vaccine that had already been administered instead of the new one intended.*
		Heavy workload due to a personnel shortage (31, 5%)	*The doctor mentioned that she couldn't prescribe the necessary antibiotics on time due to understaffing, leading to the spread of the patient's infection.*
	Communication deficiencies between doctor and patient (68, 12%)	General communication challenges at the doctor's office (58, 10%)	*The patient stopped the tetracycline course too early due to a misunderstanding of the doctor's instructions to take only a few tablets given from the hospital.*
		Doctor's inadequate language skills (6, 1%)	*The antibiotic was prescribed with the instructions “3.2 ml orally if necessary, single dose no more than 2 ml.” Due to limited language skills, the doctor confused regular and as-needed dosing. The treatment would likely have failed without the pharmacist clarifying the dosage instructions.*
		The language barrier between doctor and patient (4, 1%)	*The customer did not understand the instructions given in the emergency room regarding where to pick up the child's antibiotic and how to use it, which delayed treatment.*
	Deficiencies in the information flow in the organization (57, 10%)	From doctor to patient (40, 7%)	*The doctor had prescribed a special permit*a-h*medicine without seeing the patient and had not explained the special permit process or the duration. As a result, the patient arrived too late to pick up the medication, and the start of the medication treatment was delayed.*
		From nurse to doctor (10, 2%)	*The nurse was responsible for notifying the doctor about the warfarin INR monitoring results to initiate temporary administration of the injectable blood thinner alongside the warfarin. However, a communication breakdown between the nurse and the doctor delayed administering the blood thinner for several days.*
		From doctor to nursing home (7, 1%)	*Alprazolam medication was inadvertently continued when the nurse did not receive instructions to discontinue it from the doctor.*
	Operating practices in the organization (36, 6%)	Unclear prescription renewal procedures (15, 3%)	*The doctor renewed the metformin prescription for a short-acting tablet instead of the long-acting one, causing nausea and stomach symptoms for the patient.*
		Unclear discharge situation at the hospital (10, 2%)	*The antiepileptic drug levetiracetam was stopped after discharge from the hospital because the patient was not informed at the time of discharge that the medication should also be continued at home.*
		Unorganised prescription writing schedule (8, 1%)	*The prescription for the antibiotic to treat an infection was forgotten, causing the patient to endure pain while waiting for treatment.*
		Patient treated by several different doctors (3, 0,5%)	*The patient had been prescribed cortisone for long-term use. Later, another doctor prescribed a calcium and vitamin D supplement and a stomach protection drug to be taken alongside cortisone. However, the patient did not understand that these new medications were meant to be taken immediately. At the next doctor's visit, a different doctor was unaware of the additional medications prescribed by the previous doctor. As a result, the patient went without the appropriate medicines for more than half a year during the cortisone treatment, leading to various side effects.*
	Availability of information in the organization (21, 4%)	Incomplete available patient data (8, 1%)	*The patient had been injected with the denosumab (Xgeva) too frequently at the nurse's office due to insufficient patient information.*
		Information system deficiency (8, 1%)	*The interchangeability of short- and long-acting forms of metformin differed between the doctor's information system and Fimea's list of interchangeable preparations, leading to a prescribing error.*
		Unknown use of other medications by the patient (5, 0,9%)	*The doctor was unaware of the patient's use of antiarrhythmic medication (flecainide) when prescribing a psychotropic drug that had a category D interaction with the antiarrhythmic drug.*
	The education and employee orientation (19, 3%)	Potential deficiencies in prescription-writing skills (11, 2%)	*An incorrect iteration*a-h*resulted in the excessive use of zolpidem.*
		Insufficient training of a doctor or nurse (8, 1%)	*The newly graduated doctor's inadequate orientation delayed the start of the patient's anticoagulant medication because the doctor lacked sufficient understanding of how to write a prescription for reimbursement.*
	Unclear or missing instructions (16, 3%)	Incorrect or missing mark for reimbursementa-h (7, 1%)	*Rivaroxaban was prescribed incorrectly without the correct reimbursement mark. The pharmacy advised the doctor to prescribe it correctly for reimbursement, preventing a delay in starting the medication.*
		Missing instructions for ex temporea-h prescription writing (6, 1%)	*The prescription was missing the strength and dosage of doxepin.*
		Missing guidance for prescribing without a personal identification numbera-h (2, 0,4%)	*The start of the antibiotic treatment was delayed because the doctor did not have instructions on prescribing it to a patient without a personal identification number.*
		Unclear instructions for documenting a minor's capacity for independent decision-making in prescriptionsa-h (1, 0,2%)	*The child's vaccination was delayed because the doctor did not have instructions on how to mark the minor's decision-making capacity on the prescription, making the prescription inaccessible to the guardian.*

Tasks			
	Work methods (88, 15%)	Incorrect prescription had not been invalidated (30, 5%)	*The doctor had lowered the patient's metformin dosage but also left the prescription with the old dosage in effect, leading to the patient taking an overdose of 5 g of metformin per day and suffering from severe diarrhea and nausea until the pharmacy intervened the error.*
		Unclear principles of using the “Sic!” marka-h (30, 5%)	*The lack of “*Sic*!” mark resulted in uncertainty in the pharmacy regarding the appropriateness of the morphine solution prescribed to a patient with lung disease for shortness of breath*.
		Unfilled dosage instruction (15, 3%)	*The dosage instruction was left in the form of”tablet times a day.”*
		The prescription does not correspond to the dosage change given verbally (13, 2%)	*The doctor had adjusted the patient's asthma medication dosage and communicated this information verbally. However, the patient started questioning what had been communicated with the doctor at the pharmacy. The patient was pregnant and considered dosage accuracy even more crucial, yet no changes had been made to the written prescription.*

Person			
	Knowledge and skills (48, 8%)	Doctor's inadequate use of available information (15, 3%)	*The doctor prescribed an antibiotic that was not generally available through community pharmacies but could only be obtained from a hospital supply, causing a delay in the start of the treatment.*
		Potential deficiencies in doctor's knowledge on dosing aspects (23, 4%)	*The doctor prescribed asthma medication to be taken with the wrong dosing devices, potentially preventing proper use of the medication.*
		Deficiencies in doctor's knowledge in drug-drug interactions (5, 0,9%)	*The doctor was unaware of the category D interaction between clozapine and the prescribed antibiotics (ciprofloxacin and trimethoprim).*
		Deficiencies in doctor's knowledge in medication-specific prescribing conditionsa-h (5, 0,9%)	*The doctor prescribed clozapine without knowing the prescription condition for a maximum of one month's worth of medicine due to the high risk of side effects.*
	Individual actions within prescribing (25, 4%)	Potential negligence situations (25, 4%)	*The doctor initially prescribed too little medication, and when the pharmacy requested a correction, the doctor prescribed an eardrop for the eye. Later, the doctor apologized for carelessness.*

Care processes			
	Points of risk in the automated dose dispensing process (20, 4%)	The updated medication list has not been sent to the pharmacy (11, 2%)	*Anti-epileptic medicine clobazam was at risk of being left out of the dispensing, but the pharmacy had noticed a discrepancy between the prescription and the medication list.*
		Missing or incorrect prescription for the dose dispensing (3, 0,5%)	*Venlafaxine was at risk of being dispensed in too small a dose, but the pharmacy had noticed the discrepancy between the prescription and the medication list.*
		Outdated list of medications (2, 0,4%)	*Apixaban was dispensed in double the amount needed for 2 months because the medication list contained an outdated dose of 5 mg × 1 instead of 2.5 mg × 1.*
		A course-based medication remained in use due to a medication mix-up (2, 0,4%)	*Pantoprazole was used for two years although the present duration was intended for the cortisone course of the patient.*
		A disrupted care pathway between doctor and nursing home (2, 0,4%)	*Amlodipine was dispensed at an excessive dose because information about the change had not been communicated through the care pathway.*

Medicinal product			
	Packaging and other features (11, 2%)	Look-alike medicine (9, 2%)	*The patient was mistakenly given short-acting oxycodone instead of the intended long-acting prescription, resulting in a deterioration of the patient's pain management.*
		Sound-alike medicine (2, 0,4%)	*The medicines prednisone and prednisolone were mixed up when the prescription was written.*

Technologies and tools			
	Technical problem (6, 1%)	Technical problem in prescription writing (6, 1%)	*The doctor did not renew the patient's prescription for denosumab because she couldn't find the original prescription due to a technical problem with the prescription-writing program, leading to a delay in the injection.*

Environment			
	Physical environment (1, 0,2%)	Noise (1, 0,2%)	*Due to the disruptive noise from the renovation, the doctor mistakenly prescribed isosorbide mononitrate tablets at a higher dose, intending to prescribe isosorbide dinitrate spray.*

n = 573, indicating the total frequency of contributing factors mentioned in reports on incidents originating in social and healthcare organisations.

⁎ Quotations from the narrative descriptions of the incident reports to demonstrate the logic of the content analysis.

a-h See Additional file 1 for the terminology in the table on special features in the Finnish prescribing process.

**Fig. 4:**
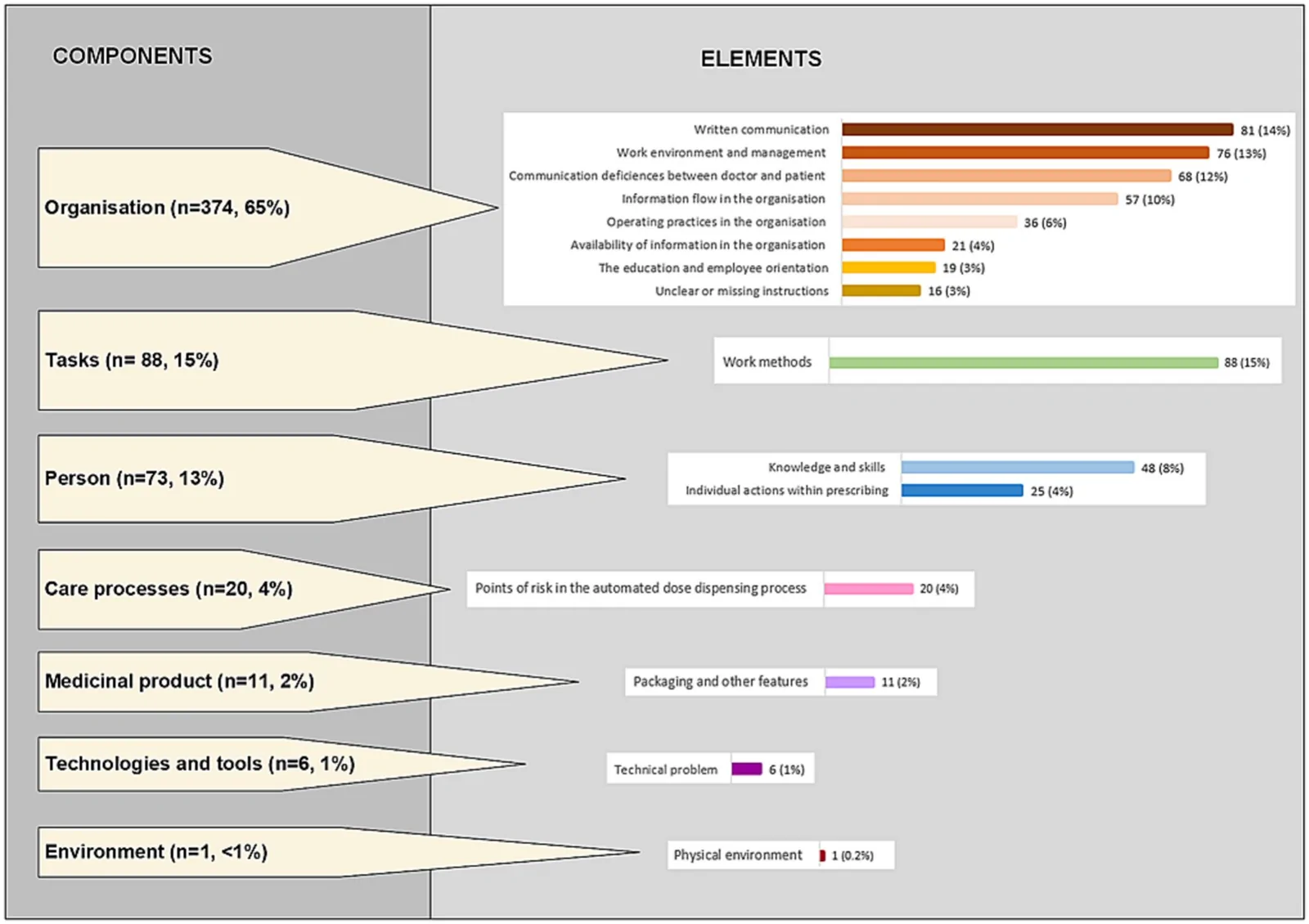
Groups of contributing factors (elements) represented under components based on the Systems Engineering Initiative for Patient Safety (SEIPS) model.^31^ Numbers (n) and percentages (%) indicate the frequency of each element and component in the data (*n* = 573, i.e., the total frequency of contributing factors mentioned in medication incident reports originating in social and healthcare organisations).

## 3 Results

Contributing factors were identified in 367 (79,6%) of the 461 incident reports. Across these reports, contributing factors were reported a total of 573 times (Fig. 4). Most incident reports (*n* = 218/461, 59%) involved a single contributing factor. In comparison, 95 cases (26%) had two contributing factors, and 40 cases (11%) had three. The highest number of contributing factors identified in a single incident report was five. Four reports (1%) included five contributing factors, while 10 reports (3%) included four contributing factors. In 61 incident reports, no contributing factors were documented, indicating that the report handler omitted this information. Additionally, 33 reports explicitly stated that no contributing factors were identified, with the report handler assessing the situation and conditions in the social and healthcare unit as usual, based on the available information.

### 3.1 Organisational components

Organisational factors were the most frequently reported contributors to medication safety incidents (65%, *n* = 374/573) (Fig. 4; Table 1). The most common issues were related to communication, particularly deficiencies in written communication (14%, *n* = 81/573). In written communication, problems frequently included missing or incorrect dosing instructions, inappropriate medication selected for the patient's condition, and unclear or outdated medication lists. These shortcomings created confusion for both patients and healthcare professionals and increased the likelihood of medication errors. In addition, communication-related issues between doctors and patients were frequently identified (12%, *n* = 68/573). These included general communication challenges during consultations, language barriers, and insufficient language skills among doctors, all of which contributed to misunderstandings with potential consequences for medication safety.

Work environment and management issues (13%, *n* = 76/573) were also prominent organisational contributors (Fig. 4; Table 1). Heavy workload, workplace busyness, and limited staffing contributed to rushed decision-making and reduced opportunities to thoroughly verify prescriptions and patient information. Organisational deficiencies in information flow further affected medication safety. Problems occurred most often in the transfer of information from doctors to patients, but also between nurses and doctors, and in communication with nursing homes (e.g., the nursing home did not receive the updated dosage information after the doctor's visit, resulting in the use of an incorrect dose). Limited availability of information, such as incomplete patient data, shortcomings in information systems, and insufficient awareness of patients' other medications, complicated the existing challenges.

Less frequently detected organisational issues were related to operating practices (6%, *n* = 36/573), education and employee orientation (3%, *n* = 19/573), and system-related instructions in social and healthcare organisations (3%, *n* = 16). Examples included unclear procedures for prescription renewals and hospital discharges, unstructured routines for writing prescriptions, and risk situations caused by patients treated by multiple doctors. Insufficient prescribing skills or inadequate training among doctors and nurses were also linked to medication incidents. Unclear or missing instructions regarding specific features of the Finnish prescribing system (see Additional file 1), such as reimbursement markings, ex tempore prescribing, prescribing for patients without a personal identification number, and assessing minors' decision-making capacity, contributed to errors in medication management.

### 3.2 Tasks-related components

Work methods were the only task-related contributing factor identified (15%, *n* = 88/573), reflecting specific ways in which work practices contributed to medication incidents (Table 1). Identified issues included prescriptions that were not properly invalidated and prescriptions that did not verbally communicate dosage changes to the patient, leading to misunderstandings about the correct dosage. Unclear principles for using the “Sic!” mark (see Additional file 1) by the doctor and incomplete dosage instruction templates (templates in the patient information system that have not been systematically utilised in prescribing) further increased the risk of medication errors.

### 3.3 Person-related components

Medication safety incidents were also associated with person-related contributing factors, including doctors' knowledge and skills (8%, *n* = 48/573) and individual actions within prescribing (4%, *n* = 25/573). Individual actions were primarily associated with situations of potential negligence, whereas doctors' knowledge and skills encompassed several specific aspects [Table 1]. These included inadequate use of available information, such as failing to verify whether a medication had been withdrawn from the market or to select the appropriate dosing device, leading to delays and interruptions in medication use. Additionally, limited knowledge of dosing, drug–drug interactions, and specific prescribing conditions (see Additional file 1) contributed to medication safety incidents.

### 3.4 Care processes-related components

Care processes-related contributing factors (4%, *n* = 20/573) were identified to involve specific phases of automated dose-dispensing, highlighting vulnerabilities across different stages of the workflow. The issues comprised factors of updated medication lists not being consistently sent to the pharmacy, prescriptions for dose dispensing sometimes being missing or incorrect, and medication lists not being regularly updated. Additionally, course-based medications occasionally remained in use beyond their intended duration, and disruptions occurred in the care pathway between physicians and the nursing home. Together, these factors hindered the ability to ensure medication safety throughout the medication management and use process.

### 3.5 Other components

The least frequently observed contributing factors were associated with medicinal products, technologies, and the physical environment (Fig. 4; Table 1). These included technical issues in prescription writing, packaging, and other product-related features, such as look-alike and sound-alike (LASA) characteristics of medications, as well as environmental factors such as distracting noise during work.

## 4 Discussion

This study examined contributing factors to medication errors identified by community pharmacists to support medication risk management in social and healthcare organisations. The findings showed that organisational factors, particularly system failures, were the most frequently identified contributors to medication errors. Communication and information transfer problems, as well as human factors related to decision-making, were also commonly reported. The findings highlight the potential of community pharmacies to support system-level medication safety improvement in outpatient care. The results align with existing literature, emphasising the value of collecting such data in community pharmacies, as well as their analysis. These practices present opportunities for implementation at both national and international levels.^13^, ^22^, ^35^, ^58^ The findings support the growing recognition that community pharmacies are not only dispensing units but safety actors capable of detecting system-level weaknesses that would otherwise remain invisible in traditional reporting structures located only in care units.

The most commonly detected organisational factors, specifically system failures,^26^ included insufficient training, inadequate knowledge (e.g., updated therapeutic guidelines), workload pressures, staff shortages, a poor work environment, distractions, and interruptions. These issues compromise medication safety by increasing the risk of errors at multiple stages of the medication-use process, primarily due to elevated cognitive load and impaired concentration.^27^ Deficiencies in communication and collaboration between healthcare providers and patients, as well as limited access to comprehensive medication information, were also identified. Inadequate communication and incomplete medication records can lead to severe medication errors.^59^, ^60^ Furthermore, communication gaps between patients and doctors may reduce treatment adherence and, in turn, undermine effective medication management.^60^ Similar challenges have been reported in previous research as contributing factors to prescribing errors in inpatient settings, indicating that observations from community pharmacies provide additional insight into the factors contributing to prescribing errors in primary and outpatient care settings.^22^, ^35^ In addition to system failures, factors related to decision-making errors and other types of human error, which, according to Reason's theory, are classified as active failures, were also commonly identified.^26^ These included failures to consider risk factors (e.g., paediatric characteristics), mistakes (e.g., not taking allergies into account), and violations (e.g., incomplete prescriptions, incorrect dosing instructions). Organisational factors, however, consistently emerged as the most significant component identified in the study, facilitating the targeting of improvement measures aligned with a systems approach. This finding is also consistent with the SEIPS model,^31^ which emphasises that patient safety outcomes arise from interactions between multiple work system elements rather than from the actions of individuals alone. These findings highlight that improving medication safety in outpatient care requires not only support for individual prescribers but also addressing broader structural and environmental conditions that influence everyday clinical work, such as workflow pressures and design flaws in digital systems.

The findings support previous literature demonstrating that medication errors are multifactorial events.^1^, ^26^ Although only a single contributing factor was most commonly recorded, multiple factors often emerged across incident reports, highlighting the complexity of error occurrence. The emergence of errors is not always a straightforward or linear process, as represented in Reason's model.^16^ Similar challenges have been reported previously, with substantial variation in the documentation of contributing factors and many medication error reports lacking sufficient information to identify underlying causes.^61^ Improving the quality of error reporting in community pharmacies, particularly the identification and documentation of contributing factors, remains essential for understanding error pathways and developing effective preventive measures in outpatient care.^1^, ^6^, ^42^

### 4.1 Study limitations

Several limitations should be considered when interpreting the findings. First, contributing factors were identified by community pharmacists based on the information available to them, which may have limited the detection of all factors involved in an incident. Information from prescribers or healthcare organisations was not always available, potentially resulting in incomplete assessments. This may partly explain the substantial number of reports in which no contributing factors were identified. Second, variation in reporting quality and underreporting may have influenced the findings. Factors such as time pressure, unfamiliarity with reporting systems, limited systems-based understanding of medication safety, and an insufficiently developed safety culture may reduce both reporting frequency and reporting quality.^63^, ^64^, ^65^, ^66^ Similar challenges have been recognised internationally, as prescribing errors are known to occur more frequently than reporting data suggest.^4^, ^44^

Improving collaboration between community pharmacies and other healthcare organisations could help address some of these limitations. More accessible communication channels with prescribers and improved access to relevant patient information may support the identification of medication risks and contributing factors.^53^, ^62^ In addition, strengthening medication safety culture and promoting consistent reporting practices could improve system-wide learning and the identification of vulnerabilities that may otherwise remain undetected.

### 4.2 Future perspectives

The findings emphasise the importance of integrating community pharmacies more effectively into the broader social and healthcare system. Community pharmacies can provide unique medication safety information that complements existing reporting mechanisms and supports system-level learning. To maximise this potential, healthcare organisations should develop effective local and national processes for receiving, analysing, and utilising pharmacy-generated medication safety information. Future research should evaluate how such information is used in practice and assess its impact on medication safety outcomes. Further studies should also examine the economic implications of pharmacist-led identification and prevention of prescribing errors. Evidence on the clinical and economic benefits of these activities could support policymaking, resource allocation, and the effective integration of community pharmacies into national medication safety strategies.

## 5 Conclusion

This study identified factors contributing to prescribing errors originating from other social and healthcare organisations by analysing medication risk information reported by community pharmacists. The findings indicate that organisational and task-related factors were the main contributors to these errors, highlighting opportunities to strengthen medication safety through the implementation of preventive system-level defences. The findings provide new insights into the causes of prescribing errors in outpatient care and indicate the potential of interprofessional collaboration to support safer prescribing and medication risk management. Further research, standardised reporting and collaboration practices, and better integration of community pharmacies across the social and healthcare system are needed to advance medication safety.

## Ethics of experimentation and informed consent

The permit to use the incident data of the present study was granted by the Association of Finnish Pharmacists (the data owner). According to Finnish National Board on Research Integrity, ethical approval is not needed for retrospective register-based study unless there is a special risk for information security in merging data or it is a medical study [1]. This study was not a medical study that interfered with the patient's physical or mental integrity, as defined by the Finnish Act on Medical Studies (488/1999) [2]. The materials of the present study were received in fully anonymous format. Therefore, researchers were not able to access the identification information of anyone included in the incident report data (including patients, healthcare professionals, or other individuals). Consequently, it was not possible to acquire an informed consent from any individual involved in the reported medication incidents. This code of conduct is in accordance with the Finnish ethical guidelines of research with human participants [1]. This study was evaluated and carried out in accordance with the Declaration of Helsinki and the ethical principles of research with human participants and ethical review in the human sciences in Finland [1].

References:

[1]. Finnish National Board on Research Integrity (TENK). The ethical principles of research with human participants and ethical review in the human sciences in Finland. 2019. Available: https://tenk.fi/sites/default/files/2021-01/Ethical_review_in_human_sciences_2020.pdf

[2]. Ministry of Social Affairs and Health, Finland. Medical Research Act. No. 488/1999. Available: https://finlex.fi/api/media/statute-foreign-language-translation/235096/mainPdf/main.pdf?timestamp=1999-04-09T00%3A00%3A00.000Z

## CRediT authorship contribution statement

**Milka Lohtaja:** Writing – review & editing, Writing – original draft, Validation, Methodology, Investigation, Formal analysis, Conceptualization. **Anna Schoultz:** Writing – review & editing, Supervision, Methodology, Investigation, Formal analysis, Conceptualization. **Iina Mönkkönen:** Writing – original draft, Investigation, Formal analysis, Conceptualization. **Anna-Riia Holmström:** Writing – review & editing, Validation, Supervision, Methodology, Investigation, Formal analysis, Conceptualization.

## Funding

This research did not receive any specific grant from funding agencies in the public, commercial, or not-for-profit sectors. Open access was funded by the Helsinki University Library.

## Declaration of competing interest

The authors declare that they have no known competing financial interests or personal relationships that could have appeared to influence the work reported in this paper.

## Data Availability

The datasets analysed during the current study are not publicly available. Access to datasets needs to be negotiated separately with the organisation (the Association of Finnish Pharmacies) that owns the data.

## Appendix A Supplementary data

**Additional file 1**. Terminology for special features in the Finnish prescribing process.
