# Supplementary material for: Factors contributing to medication safety incidents observed by community pharmacists - a content analysis of incident reports
Source: Explor Res Clin Soc Pharm. 2026 Sep 21;24:100851. doi: 10.1016/j.rcsop.2026.100851 (PMC13634388; doi:10.1016/j.rcsop.2026.100851)
Supplement: Supplementary file 1 [file mmc1.docx]

**Additional file 1**. Terminology for special features in the Finnish prescribing process.

| **The term used in the study** | **Explanation of the term** |
| --- | --- |
| Special permit | The release for consumption of a medicinal product without marketing authorization in Finland by the Finnish Medicines Agency |
| Iteration of a prescription | The doctor has specified exactly how much and how often the medication can be dispensed to the patient by the pharmacy. |
| Prescribing with a correct mark for reimbursement | A prescription notation is a special clarification that the prescriber records on the prescription. The pharmacy can provide reimbursement for the medication if the prescription notation meets the required specification or pertains to a certain form or severity of the disease |
| Writing an ex tempore prescription | If no suitable medication is available, a doctor may need to prescribe an "ex tempore" medication. Instead of the name of a manufactured drug, the ex tempore prescription includes information about the ingredients and excipients of the medication to be prepared at the pharmacy. |
| Prescribing medication to a patient without a personal identification number | For patients without an official personal identification number (e.g. individuals with refugee status), prescriptions are issued using their names and dates of birth. A patient instruction sheet must always be printed for the patient to obtain the medication from the pharmacy. |
| The decision-making capacity of a minor | The assessment result of whether a minor is capable of deciding about their treatment is recorded in the patient information system each time a prescription is written or renewed. |
| The ”Sic!”-mark | The mark must be applied to the prescription when the medication has been intentionally prescribed in a manner that deviates from the dosing instructions outlined in the approved product summary characteristics. |
| Medicine-specific prescribing conditions | Some medicinal products are allowed to be prescribed only under certain conditions. If the condition is not met, the prescribing must be stopped. |
